# Dual-Tracer Assessment of Dynamic Changes in Reoxygenation and Proliferation Decrease During Fractionated Radiotherapy in Murine Tumors

**DOI:** 10.3389/fonc.2020.01046

**Published:** 2020-07-17

**Authors:** Wenjing Yu, Xiaoyu Su, Dan Zhang, Feng Qiao, Hui Wang, Jinhui Jiang, Huiqin Xu

**Affiliations:** Department of Nuclear Medicine, The First Affiliated Hospital of Anhui Medical University, Hefei, China

**Keywords:** fluoromisonidazole, fluorothymidine, hypoxia, reoxygenation, radiotherapy, PET/CT

## Abstract

**Objective:** The present work aimed to assess reoxygenation and tumor inhibition during fractionated radiotherapy (FRT) in murine tumors using ^18^F-fluoromisonidazole (^18^F-FMISO) and ^18^F-fluorothymidine (^18^F-FLT) based micro positron emission tomography/computed tomography (PET/CT).

**Materials and Methods:** A nude mouse xenograft model was established with the head and neck squamous carcinoma cell (FaDu), followed by administration of FRT. Imaging was carried out with both ^18^F-FMISO and ^18^F-FLT PET/CT, prior to FRT (Pre-FRT, 0 Gy), during FRT (Inter-FRT, 21 Gy), and after FRT (Post-FRT, 40 Gy). The maximum standardized uptake (SUVmax) and tumor-to-normal muscle ratio (TNR) were determined in regions of interest (ROIs) in ^18^F-FMISO and ^18^F-FLT PET/CT images. Then, hypoxic (HV) and proliferative tumor (PTV) volumes obtained by PET/CT were analyzed. Immunohistochemistry was performed to analyze the changes of hypoxia-inducible factor- (HIF)-1α, carbonic anhydrase 9 (CAIX), Ki67 and proliferating cell nuclear antigen (PCNA). Associations of the levels of these biomarkers with PET/CT parameters were analyzed.

**Results:** ^18^F-FMISO PET/CT demonstrated markedly elevated reduction rates of SUVmax (30.3 vs. 14.5%, *p* = 0.012), TNR (27.9 vs. 18.3%, *p* = 0.032) and HV (85.0 vs. 71.4%, *p* = 0.047) from Pre-FRT to Inter-FRT compared with values from Inter-FRT to Post-FRT. Meanwhile, PTV reduction rate in ^18^F-FLT PET/CT from Pre-FRT to Inter-FRT was significantly decreased compared with that from Inter-FRT to Post-FRT (21.2 vs. 82.7%, *p* = 0.012). Tumor HIF-1α, CAIX, Ki67, and PCNA amounts were continuously down-regulated during radiotherapy. TNR (FMISO) showed significant correlations with HIF-1α (*r* = 0.692, *p* = 0.015) and CAIX (*r* = 0.801, *p* = 0.006) amounts in xenografts, while associations of SUVmax (FMISO) with hypoxia markers were weak (*r* = 0.418, *p* = 0.041 and *r* = 0.389, *p* = 0.037, respectively). SUVmax (FLT) was significantly correlated with Ki67 (*r* = 0.792, *p* = 0.003) and PCNA (*r* = 0.837, *p* = 0.004).

**Conclusions:** Tumor reoxygenation occurs early during radiotherapy, while inhibition of cell proliferation by tumoricidal effects mainly takes place gradually with the course of radiotherapy. ^18^F-FMISO and ^18^F-FLT PET/CT are sensitive and non-invasive tools for the monitoring of tumor reoxygenation and proliferation during radiotherapy.

## Introduction

Hypoxia, commonly found in solid tumors (1), is considered to exert adverse effects on radiotherapy outcome, and shows an association with poor prognosis (2–5). Increased oxygen amounts, termed reoxygenation, is generally associated with radiation therapy. Reoxygenation is likely to play a positive role in increasing irradiation sensitivity, therefore improving tumor control (6). In order to achieve tumor reoxygenation, dose escalation in radiotherapy for hypoxic regions represents a common method. However, because radiation damages the surrounding non-cancerous tissues, the dose for a hypoxic tumor cannot exceed 200% of the standard one (7). Detection of reoxygenation and the degree of oxygen increase in tumors during fractionated radiotherapy is of great importance for dose adjustment in follow-up radiotherapy.

^18^F-fluoromisonidazole positron emission tomography/computed tomography (^18^F-FMISO PET/CT) non-invasively evaluates tumor hypoxia. Interestingly, FMISO uptake correlates with the “gold standard” of tissue measurement using invasive polarographic oxygen electrodes (8, 9), as well as pimonidazole uptake in animals with glioma (10). Our previous and several other studies have reported decreased FMISO uptake in hypoxic tumors during radiotherapy and post-treatment, indicating that reoxygenation occurs (11–16). However, cancer cells killed by irradiation no longer show FMISO uptake; thus, it is hard to correctly determine whether reduced FMISO uptake in radiotherapy results from tumor reoxygenation or reflects tumoricidal effects. In a study by Okamoto et al. (17), tumoricidal effects during radiotherapy were evaluated by assessing tumor metabolism using ^18^F-fluorodeoxyglucose (FDG) PET. However, irradiation usually causes radioactive inflammation, which can also exhibit FDG uptake. Uptake of ^18^F -FDG PET, therefore, could represent either the residual tumor or inflammation.

Apart from tumor oxygenation, another most influential factor affecting fractionated radiotherapy is the potential changes of cell proliferation, which indicates tumor activity. Fortunately, cell proliferation can be assessed by ^18^F-fluorothymidine (FLT) PET during radiation treatment. Indeed, the development of FLT as a PET tracer has enabled *in vivo* demonstration of cell proliferation (18). *In vivo* analyses suggested that FLT has higher tumor specificity than FDG, and can distinguish tumor tissue from inflammation (19, 20). Tumor cells with low FLT and FMISO uptake levels are considered to be inactive and will undergo death. Meanwhile, those with high FLT and low FMISO levels are active with no hypoxia.

In the current study, using an experimental murine tumor model, we investigated tumor reoxygenation and tumor proliferation changes during radiotherapy with ^18^F-FMISO PET/CT and ^18^F-FLT PET/CT prior to, during, and following fractionated radiotherapy (FRT), with the aim to detect the relationship between tumor reoxygenation and tumoricidal effects during radiotherapy.

## Materials and Methods

### Establishment of Tumor Model

All experimental studies were approved by the Institute of Anhui Medical University, and followed AAALAC and IACUC guidelines. The head and neck squamous carcinoma cell line (FaDu) was from the Anhui Medical University animal center. Four to five weeks old female BALB/c nude mice (20–25 g), underwent anesthesia with 1% isoflurane and received a subcutaneous injection of 5.0 × 10^6^ cells in 0.2 mL phosphate-buffered saline (PBS) into the right flank. Tumors of 6–7 mm in diameter (10 days after injection) were selected for experiments. Tumor diameters were measured every day, and gross tumor volume (GTV) was derived as: V (cm^3)^ = length (cm) × width^2^ (cm^2^) × 0.5.

### Irradiation of Tumors

Tumor-bearing mice were divided into two groups: (i) control group (*n* = 5) did not receive any treatment; (ii) IR group (*n* = 16) was exposed to 3 Gy daily to a maximum dose of 40 Gy with a VARIAN 23 EX medical linear accelerator (Varian Medical Systems, USA). The tumor-bearing mice were lightly anesthetized with 1% isoflurane and placed in a circular irradiation jig. Then, the tumor-bearing legs were gently extended into the central part of the jig, taped, and the animals were covered with a 3-mm-thick lead sheet. The irradiation factors were 6 mV, 6/100 Varian linear accelerator at a dose rate of 200 mU/min.

### PET/CT Imaging

All mice were scanned with both ^18^F-FMISO and ^18^F-FLT PET/CT prior to (Pre-FRT, 0 Gy), during (Inter-FRT, 21 Gy), and after FRT (Post-FRT, 40 Gy). When reached a dose of 21Gy, radiotherapy was break for 2 days for Inter-FRT imaging. ^18^F-FMISO and ^18^F-FLT PET/CT scan (Inveon, Siemens, Micro PET research center of shanghai Ruijin hospital) were conducted on 2 consecutive days. Both ^18^F-FMISO and ^18^F-FLT were provided by the molecular imaging center of Shanghai Xinhua hospital (China), with radiochemical purity above 95% to qualify for use in imaging. For PET/CT, one dose of 5.55 MBq (150 μCi) of ^18^F-FMISO or ^18^F-FLT was intravenously administered to each mouse via the tail vein. PET was performed 2 h following ^18^F-FMISO and 1 h upon ^18^F-FLT administration, and image reconstruction was carried out with the Inveon Acquisition Workplace software v2.0 (Siemens Preclinical Solutions). CT-guided percutaneous biopsy was performed for pathological study at Pre-FRT and Inter-FRT while all mice were sacrificed for pathological analysis at Post-FRT.

### Image Analysis

To evaluate tumor hypoxia, maximum standardized uptake (SUVmax) and the tumor-to-normal muscle ratio (TNR) were used in this study for quantitative assessment of ^18^F-FMISO PET/CT data. SUVmax was determined by assessing the maximal radioactivity in a region of interest (ROI). The TNR was derived as the tumor's SUVmax divided by the SUVmax of non-cancerous tissue. Hypoxia volume (HV) was the volume with TNR ≥1.25 (21). Tumor reoxygenation was analyzed according to TNR and HV changes. To assess tumor proliferation, we measured the tumor's SUVmax and calculated the proliferation target volume (PTV) in the ^18^F-FLT PET/CT image. The PTV was the volume with SUVmax >1.4 as previously reported (22). In ^18^F-FMISO and ^18^F-FLT PET/CT images, reduction rates from Pre-FRT to Inter-FRT were derived as (uptake in Pre-FRT—uptake in Inter-FRT)/uptake in Pre-FRT.

### Pathological Assays

Tumor specimens underwent formalin fixation, paraffin-embedding, and sectioning at 4 μm for immunohistochemical staining. Hypoxia (hypoxia-inducible factor- [HIF-] 1α and carbonic anhydrase IX [CAIX]) and proliferation (the proliferation antigen Ki67 and proliferating cell nuclear antigen [PCNA]) markers were evaluated immunohistochemically. Antibodies raised against HIF-1α (1/200; Novus Biologicals, USA), CAIX (1/400; Santa Cruz, USA), Ki67 (1/500; Zhongshan Jinqiao, China) and PCNA (1/400, Santa Cruz) were probed. HIF-1α, CAIX, Ki67 and PCNA levels were obtained by counting stained cells in 10 randomly selected high-power fields (×400), and graded as follows based on the percentages of cells stained: 0+, 0–10%; 1+, 10–25%; 2+, 25–50%; 3+, 50–75%; 4+, >75%.

### Statistical Analysis

All statistical analyses were performed with SPSS v17.0. Data are mean ± SD. Changes in SUVmax, TNR, HV, PTV and the expression levels of biomarkers Pre-FRT, Inter- FRT, and Post-FRT were analyzed by *t*-test. Associations of SUVmax, TNR and tumor volume with the above biomarkers were assessed by Pearson correlation analysis. *P* < 0.05 indicated statistical significance.

## Results

### Gross Tumor Volume (GTV) Change

The preliminary studies were performed to maintain animal welfare and tolerance to the tumor and irradiation. However, even under the daily 2 Gy dose, owing to the continual increase in tumor volume, many tumors grew to exceed 8 or 10 mm in diameter. Consequently, the tumor ulceration and infection became issues of concern, and the increase in the abundance of necrotic tissue in the tumor may affect the detection of hypoxic tissue. Therefore, we increased the daily dose as 3 Gy and started the tumor radiotherapy when the diameter of the lesion was 6–7 mm to prevent the tumors from reaching the 10-mm ulceration threshold.

**Figure 1:**
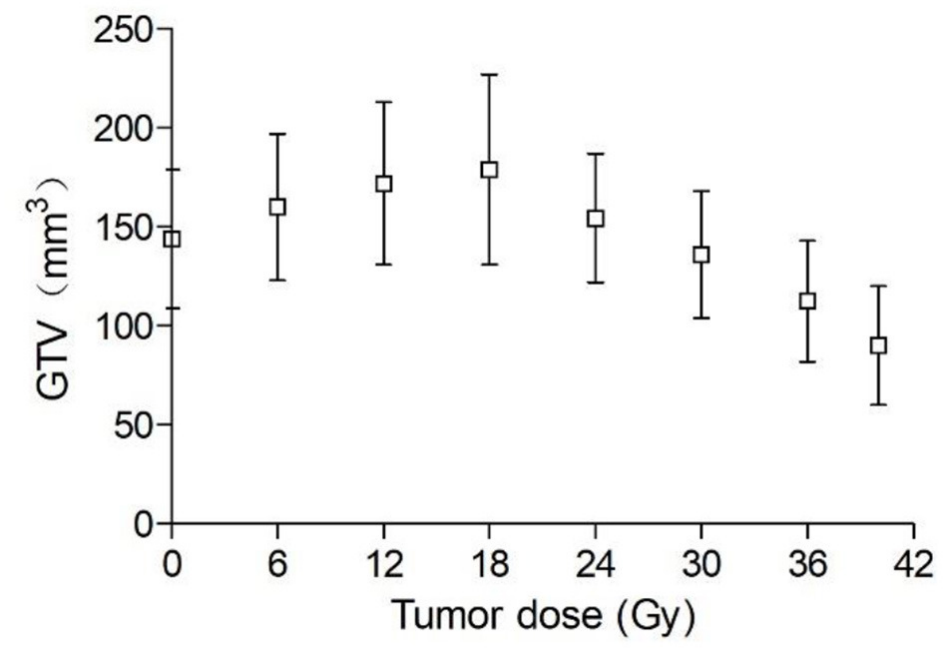
Changes in GTV during radiotherapy (maximum dose 40 Gy) (*n* = 16).

Ten days following tumor cell administration, the 16 animal models in IR group assessed showed overt subcutaneous tumors on the right hind flank. The tumor average diameter was 6.8 ± 0.4 mm pre-irradiation, corresponding to an average volume of 144 mm^3^. As shown in Figure 1, GTV increased during the first week of irradiation and began to decrease only after 18 Gy (6th day of radiotherapy). GTV increased on average by 24.3% from pre-irradiation (144 ± 29 mm^3^) to a cumulative dose of 18 Gy (179 ± 46 mm^3^), with a mean overall decrease of 45% by treatment end (50 Gy; 90 ± 18 mm^3^). Conversely, owing to the continual increase of GTV in the control group, some tumors grew beyond 10 and 12 mm in diameter. Thus, some of the tumor mice in the control group were sacrificed early for animal welfare.

### PET/CT Data

**Figure 2:**
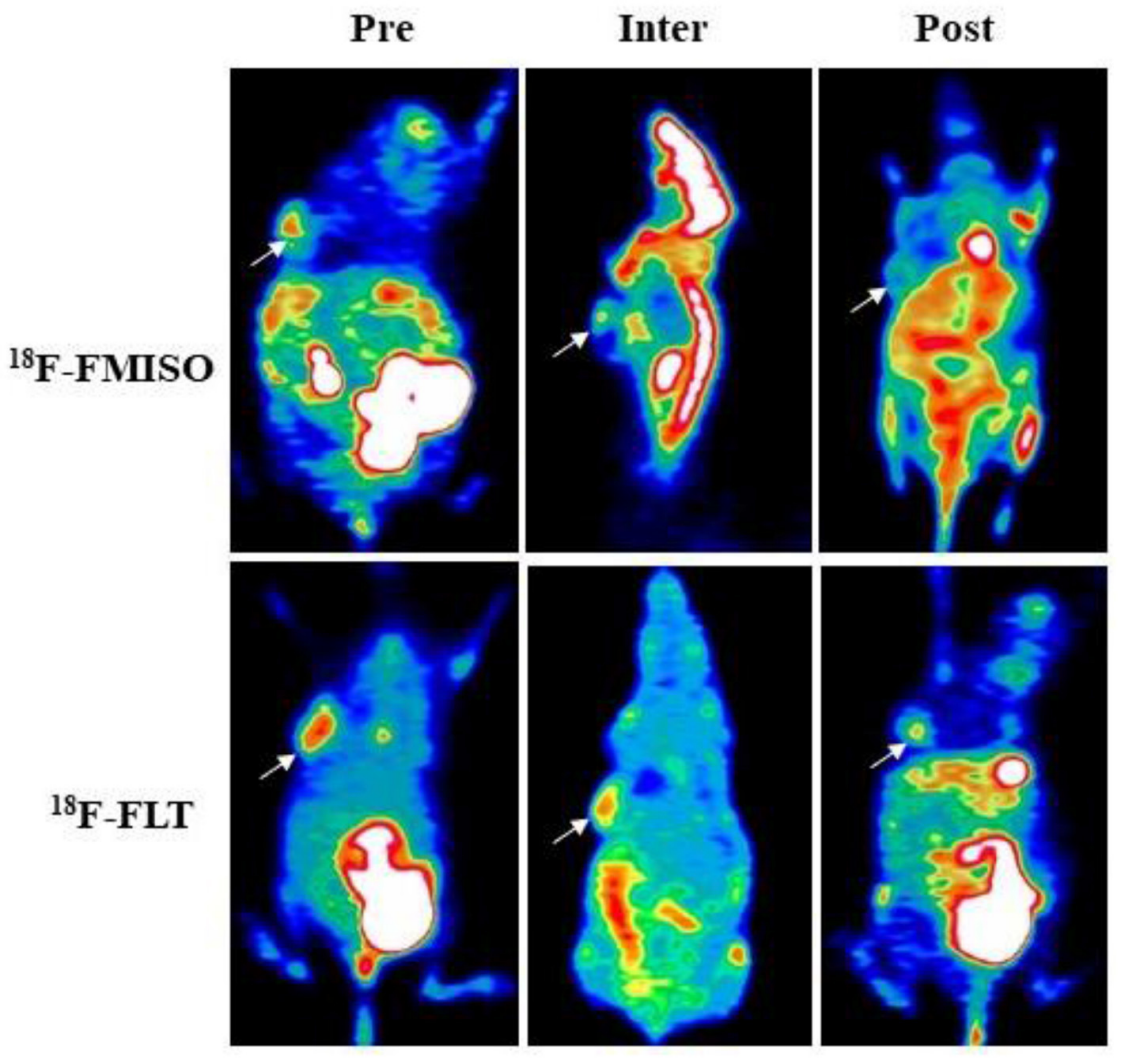
^18^F-FMISO and ^18^F-FLT-based micro-positron emission tomography (PET) detecting FaDu xenografts. White arrows indicate tumor location (Pre, Inter and Post represent data before (0 Gy), during (21 Gy), and after (40 Gy) radiotherapy, respectively). In ^18^F-FMISO PET images, the TNR, SUVmax, and HV were significantly decreased from Pre-FRT to Inter-FRT (*p* < 0.001, *p* < 0.001, *p* = 0.031, respectively) and from Inter-FRT to Post-FRT (*p* = 0.004, *p* = 0.008, *p* = 0.025, respectively). In ^18^F-FLT PET images, SUVmax and PTV were significantly decreased from Pre-FRT to Inter-FRT (*p* = 0.032, *p* = 0.041, respectively) and from Inter-FRT to Post-FRT (*p* = 0.017, *p* < 0.001, respectively).

**Figure 3:**
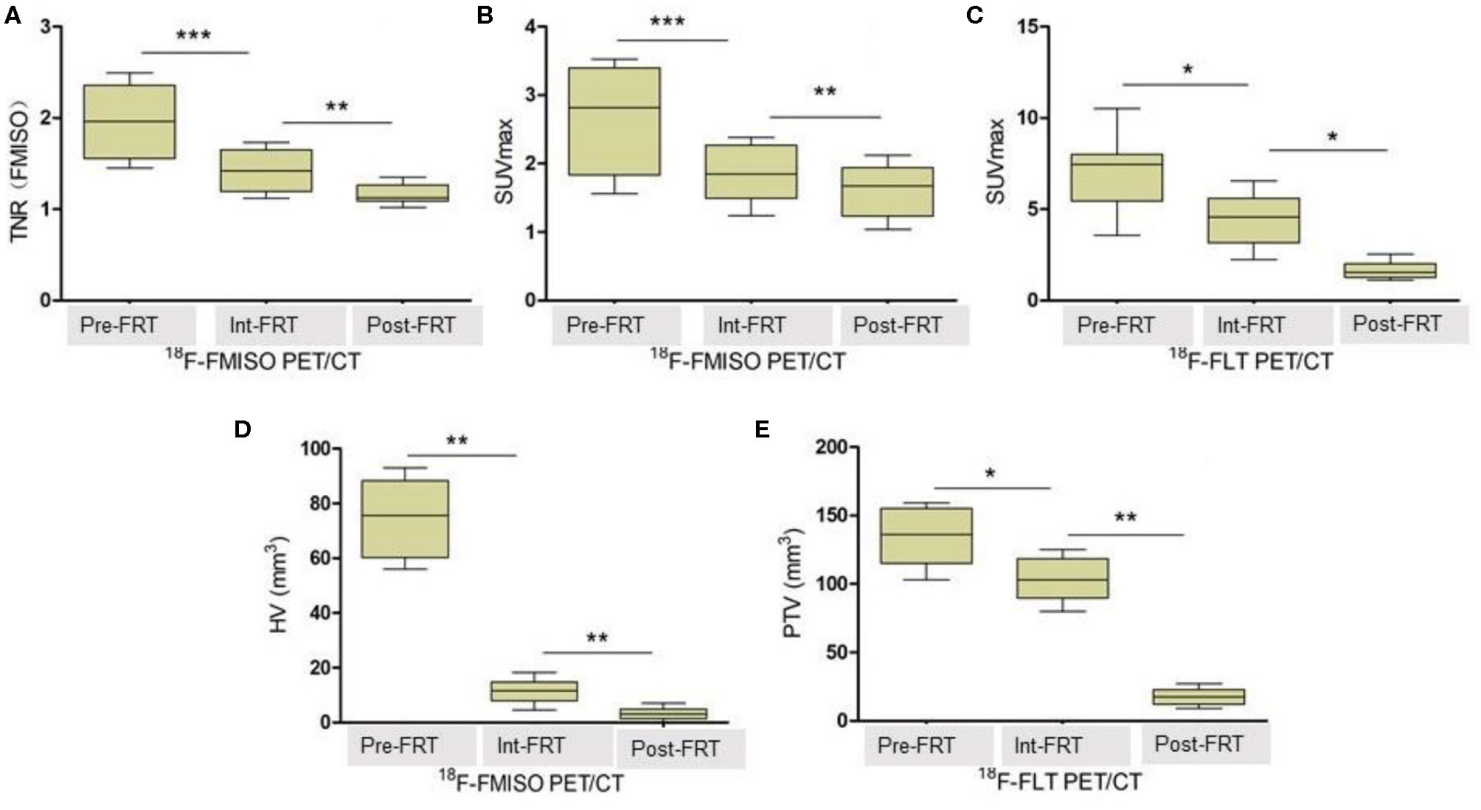
TNR, SUVmax, HV and PTV changes detected by ^18^F-FMISO PET/CT and ^18^F-FLT PET/CT, respectively. **(A)** Changes of TNR (FMISO) detected by ^18^F-FMISO PET/CT during radiotherapy. **(B)** Changes of SUVmax detected by ^18^F-FMISO PET/CT during radiotherapy. **(C)** Changes of SUVmax detected by ^18^F-FLT PET/CT during radiotherapy. **(D)** Changes of HV detected by ^18^F-FMISO PET/CT during radiotherapy. **(E)** Changes of PTV detected by ^18^F-FLT PET/CT during radiotherapy. The reduction in all parameters was significant. *P < 0.05, **P < 0.01, ***P < 0.01 by Student's *t*-test (*n* = 16). TNR, tumor-to-normal muscle ratio of maximum radiotracer uptake; HV, hypoxic volume; PTV, proliferative tumor volume; FRT, fractionated radiotherapy.

As shown in Table 1, both ^18^F-FMISO-SUVmax and ^18^F-FMISO-TNR were markedly reduced from Pre-FRT to Post-FRT in the IR group. In addition, the reduced rates of SUVmax and TNR from Pre-FRT to Inter-FRT (30.3 and 27.9%, respectively) were significantly higher than those from Inter-FRT to Post-FRT (14.5 and 18.3%, respectively). For ^18^F-FLT PET/CT, ^18^F-FLT-SUVmax was significantly and continually decreased from Pre-FRT to Post-FRT; however, the reduction rate from Pre-FRT to Inter-FRT (36.8%) was similar to that from Inter-FRT to Post-FRT (51.0%; *p* = 0.087). Both HV and PTV were significantly decreased from Inter-FRT to Post-FRT. The mean reduction rate of HV from Pre-FRT to Inter-FRT (85%) was significantly elevated as compared to that from Inter-FRT to Post-FRT (71.4%), while the PTV reduction rate from Pre-FRT to Inter-FRT (21.2%) was remarkably lower as compared to that from Inter-FRT to Post-FRT (82.7%) (Figures 2, 3). Conversely, all the above PET parameters in the control group showed a significant continual increase during the study, which was due to the increased oxygen depletion and rapid tumor proliferation.

**Table 1:** Uptake changes detected by ^18^F-FMISO and ^18^F-FLT PET/CT at Pre-FRT, Inter-FRT, and post-FRT.

	**Pre-FRT**	**Inter-FRT (*p*-value)**	**Post-FRT (*p*-value)**	**Reduction rate from pre- to inter-FRT**	**Reduction rate from inter- to post-FRT (*p*-value)**
**IR group (*****n*** **=** **16)**					
^18^F-FMISO-SUVmax	2.67 ± 0.78	1.86 ± 0.41 (*p* < 0.001)	1.59 ± 0.39 (*p* = 0.008)	30.3 ± 21.6%	14.5 ± 10.0% (*p* = 0.012)
^18^F-FMISO-TNR	1.97 ± 0.40	1.42 ± 0.23 (*p* < 0.001)	1.16 ± 0.11 (*p* = 0.004)	27.9 ± 18.2%	18.3 ± 9.8% (*p* = 0.032)
^18^F-FLT-SUVmax	7.01 ± 3.10	4.43 ± 2.45 (*p* = 0.032)	1.80 ± 1.37 (*p* = 0.017)	36.8 ± 28.22%	51.0 ± 38.2% (*p* = 0.087)
^18^F-FMISO-HV (mm^3^)	74.53 ± 18.20	11.20 ± 6.79 (*p* = 0.031)	3.27 ± 4.12 (*p* = 0.025)	85.0 ± 28.2%	71.4 ± 26.3% (*p* = 0.047)
^18^F-FLT-PTV (mm^3^)	132.00 ± 27.00	104.00 ± 21.00 (*p* = 0.041)	18.40 ± 9.70 (*p* < 0.001)	21.2 ± 14.2%	82.7 ± 48.4% (*p* = 0.012)
**Control group (*****n*** **=** **5)**					
^18^F-FMISO-SUVmax	2.31 ± 0.45	2.66 ± 0.78 (*p* < 0.001)	2.98 ± 0.56 (*p* < 0.001)	−15.2 ± 11.1%	−12.0 ± 7.4% (*p* = 0.034)
^18^F-FMISO-TNR	1.62 ± 0.31	1.89 ± 0.43 (*p* < 0.001)	2.12 ± 0.46 (*p* < 0.001)	−16.7 ± 12.1%	−12.2 ± 6.7% (*p* = 0.041)
^18^F-FLT-SUVmax	6.35 ± 3.15	9.78 ± 4.76 (*p* < 0.001)	11.79 ± 5.37 (*p* < 0.001)	−54.0 ± 21.43%	−20.6 ± 13.2% (*p* < 0.001)
^18^F-FMISO-HV (mm^3^)	68.21 ± 15.40	81.12 ± 18.77 (*p* < 0.001)	99.33 ± 24.52 (*p* < 0.001)	−18.9 ± 10.77%	−22.4 ± 14.3% (*p* = 0.004)
^18^F-FLT-PTV (mm^3^)	137.00 ± 32.00	198.00 ± 57.45 (*p* < 0.001)	252.11 ± 71.46 (*p* < 0.001)	−44.5 ± 25.1%	−27.3 ± 17.3% (*p* < 0.001)

Of all the tumor-bearing mice in the IR group, five tumors showed hypoxia during Inter-FRT FMISO PET, which persisted Post-FRT in one mouse. Interestingly, decreased HV could render these lesions highly resistant to radiotherapy. Furthermore, nine tumor-bearing mice showed strong proliferation at Inter-FRT FLT PET, and the positive uptake persisted Post-FRT in four mice.

### Expression Levels of Hypoxia and Proliferation Markers

**Figure 4:**
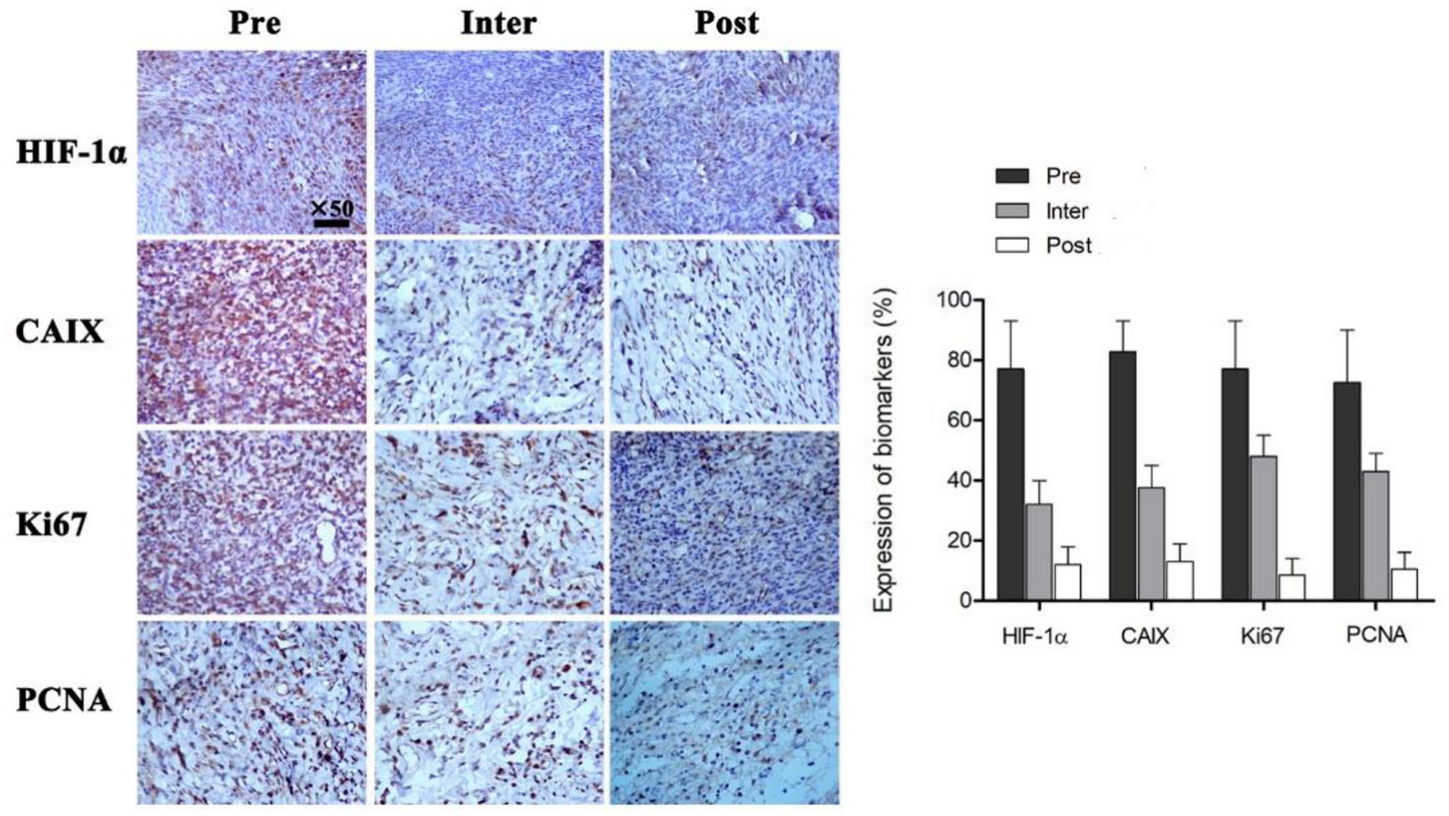
Immunohistochemical assessment of multiple tumor biomarkers before, during and after radiotherapy. All the indicated biomarkers in tumors were continuously downregulated during irradiation. *P* < 0.05 by Student's *t*-test (*n* = 16). HIF-1α, hypoxia-inducible factor; CAIX, carbonic anhydrase 9; Ki67, proliferation antigen; PCNA, proliferating cell nuclear antigen.

Pathological examination was performed Pre (at 0 Gy), Inter (at 21 Gy) and Post (at 40 Gy) radiotherapy (Figure 4). Immunohistochemical assays were carried out to evaluate tumor hypoxia (HIF-1α, CAIX) and proliferation (Ki67, PCNA) markers. There were 77.63 ± 15.96, 32.76 ± 8.47 and 11.33 ± 5.71% cells with positive HIF-1α staining at Pre-FRT, Inter-FRT and Post-FRT, respectively. Cells expressing CAIX at Pre-FRT, Inter-FRT and Post-FRT accounted for 82.56 ± 10.33, 37.32 ± 7.28, and 13.12 ± 6.12%, respectively. There were 77.54 ± 16.42, 48.76 ± 7.24, and 8.31 ± 6.12% cells expressing Ki67 at Pre-FRT, Inter-FRT and Post-FRT, respectively. PCNA at Pre-FRT, Inter-FRT and Post-FRT was expressed by 72.96 ± 18.49, 43.11 ± 6.77 and 10.12 ± 5.82% cells, respectively. These tumor biomarkers showed significant differences between Pre-FRT and Inter-FRT (*p* < 0.05), and between Inter-FRT and Post-FRT (*p* < 0.05). These findings suggested hypoxia and proliferation markers were continuously down-regulated during radiotherapy, which was consistent with PET imaging data.

### Associations of Uptake of Radiotracers With Tumor Volume (HV, PTV and GTV)

Both SUVmax (FMISO) and TNR (FMISO) were significantly associated with HV (*r* = 0.686, *p* = 0.024 and *r* = 0.763, *p* = 0.016, respectively), but showed no associations with GTV (*r* = 0.325, *p* = 0.178 and *r* = 0.145, *p* = 0.216, respectively). SUVmax (FLT) was significantly correlated with PTV (*r* = 0.842, *p* = 0.009), but showed no correlation with GTV (*r* = 0.211, *p* = 1.435) (Table 2).

**Table 2:** Correlation analysis between PET tracers and tumor biological parameters.

		**HV**	**PTV**	**GTV**	**HIF-1α**	**CAIX**	**Ki67**	**PCNA**
SUVmax (FMISO)	Pearson correlation	0.686^*^	0.215	0.325	0.418^*^	0.389^*^	0.237	0.112
	Sig(2-tailed)	0.024	0.468	0.178	0.041	0.037	0.745	0.253
	N	16	16	16	16	16	16	16
TNR (FMISO)	Pearson correlation	0.763^*^	0.341	0.145	0.692^*^	0.801^**^	0.412	0.215
	Sig (2-tailed)	0.016	0.842	0.216	0.015	0.006	0.341	0.547
	N	16	16	16	16	16	16	16
SUVmax (FLT)	Pearson correlation	0.308	0.842^**^	0.211	0.304	0.523	0.792^**^	0.837^**^
	Sig (2-tailed)	0.264	0.009	1.435	0.271	0.222	0.003	0.004
	N	16	16	16	16	16	16	16

* P < 0.05;

** *P < 0.01*.

### Associations of Uptake of Radiotracers With Tumor Markers

TNR (FMISO) was significantly correlated with hypoxia markers, including HIF-1α and CAIX (*r* = 0.692, *p* = 0.015 and *r* = 0.801, *p* = 0.006, respectively). Meanwhile, weak correlations were found between SUVmax (FMISO) and hypoxia markers (*r* = 0.418, *p* = 0.041 and *r* = 0.389, *p* = 0.037, respectively). No significant associations were detected of FMISO uptake with proliferation markers. SUVmax (FLT) was significantly correlated with Ki67 and PCNA (*r* = 0.792, *p* = 0.003 and *r* = 0.837, *p* = 0.004, respectively). SUVmax (FLT) was not associated with hypoxia markers (Table 2).

## Discussion

Radiotherapy has been used extensively to treat cancer patients because it damages most solid tumors without penetration limits. It is well-known that the “four R's” (repair of DNA damage, redistribution of cells in the cell cycle, repopulation, and reoxygenation of hypoxic tumor areas) play a role in radiotherapy. In this study, we mainly explored reoxygenation and tumor proliferation during radiotherapy. Among the “four R's,” reoxygenation may be the most important in fractionated radiotherapy (23). A hypoxic microenvironment in solid tumors leads to severe radioresistance, which results in poor efficacy of radiotherapy. The maximal benefit of fractionation against tumors is attributed to the reoxygenation of surviving hypoxic cells before the next fraction. The assessment of tumor hypoxia during treatment discriminates between radiosensitive and radioresistant tumors, and hence, the application of doses in different areas can be optimized to maximize the tumor damage and protect normal tissue.

In the current study with head and neck squamous carcinoma (FaDu) xenograft model, serial ^18^F-FMISO and ^18^F-FLT PET/CT scans showed tumor reoxygenation and gradual decline in tumor cell proliferation during radiotherapy. FMISO uptake was reduced earlier than FLT uptake during radiotherapy. The reduction rates of FMISO uptake and HV at the beginning of radiotherapy (from 0 to 21 Gy) were significantly higher than those observed in the late phase of treatment (from 21 to 40 Gy). However, the decrease of FLT uptake gradually with the course of radiotherapy. Compared with the significant reduction of hypoxia, SUVmax (FLT) and PTV were still at relatively high levels at Inter-FRT. These findings support our previous hypothesis that “the tumor is still active, but not hypoxic.” In case such assumption holds, the current data suggest that the amounts of hypoxic cells are reduced earlier than those of active cells during fractionated radiation treatment. In other words, reduction in FMISO uptake in the early phase indicates tumor reoxygenation rather than tumoricidal effects.

In this study, HV was significantly decreased in Inter-FRT ^18^F-FMISO PET/CT at 21 Gy; meanwhile, PTV reduction in the early phase of treatment was markedly less pronounced than that of HV. It is well-known that tumor cells with hypoxia are more radiation-resistant than oxygenated counterparts (24, 25). Thus, hypoxic cancer cells are not likely killed earlier by irradiation compared with non-hypoxic ones. More likely, cells with elevated FMISO uptake in the HV pre-radiotherapy turned into cells with reduced FMISO uptake. This corroborated a previous study of patients with primary head-and-neck cancer by Okamoto et al. (17).

In addition, the FMISO uptake is continued in five tumor-bearing mice (31.2%, 5/16) at Inter-FRT and one tumor-bearing mouse (6.2%, 1/16) at Post-FRT. FMISO PET during radiotherapy may contribute to the detection of such highly radioresistant tumors. Clinically, this phenomenon indicated that additional chemotherapy or molecular targeted therapy might be needed for these patients.

Reoxygenation occurred early after radiotherapy initiation as shown above; this is consistent with previous reports assessing other xenograft tumor cell lines or *in vivo* tumors (5, 26). However, Harriss et al. (6) reported that reoxygenation occurs until near the end of irradiation treatment, based on pO_2_ measurements using the OxyLab probe. There are many possibilities which could explain such discrepancy. Tumor oxygenation depends on multiple individual features such as the amounts of cells under hypoxia, the extent of oxygen elevation, and the timing/progression of such effect during treatment (27, 28). Tumor type, volume and vascularization, as well as the structure and pressure of neighboring tissues are influential factors for reoxygenation initiation (6). As a consequence, pre-treatment evaluation of tumors is critical if individualized radiotherapy is to be implemented.

Mice underwent exposure to 3 Gy daily in this study. As shown in Figure 1, GTV was increased during the first week of radiotherapy, and began to decrease only after a cumulative dose of 18 Gy. In contrast, HV and PTV decreased gradually with increasing radiotherapy time. These findings indicate that tumor volume has no sensitivity for early efficacy assessment. Gross tumor volume is influenced by various factors such as fibrosis components, infiltration of inflammatory cells and the rate at which apoptotic cancer cells are cleared (29). Thus, functional imaging should be used for early efficacy evaluation in clinic.

In order to detect changes of the microenvironment in tumor cells during radiotherapy, immunohistochemical assays were performed at Pre-FRT, Inter-FRT, and Post-FRT. Biomarkers specific to cancer are frequently employed for monitoring tumor occurrence and progression. It is admitted that the adaptation of tumor cells to the hypoxic microenvironment is regulated by HIF-1a, which is considered an independent biomarker of response to hypoxia (30). HIF-1a activation promotes the expression of CAIX, which controls intra- and extra-cellular pH homeostasis in hypoxic conditions (31). Overexpression of CAIX is correlated with poor survival/prognosis in solid tumors (32, 33). In a study by Chen et al. elevated CAIX amounts were shown to promote tumor progression (34). Furthermore, the proliferation antigen Ki67 is closely associated with cell mitosis, playing a vital role in the initiation of cell proliferation (35). Proliferating cell nuclear antigen (PCNA) is an important trigger of multiple events in DNA replication, repair and recombination in eukaryotes and archaea, thus regulating cell proliferation (36). In this study, all these biomarkers were progressively down-regulated during radiotherapy, corroborating PET/CT imaging data. Moreover, statistical analysis showed that PET/CT imaging parameters had close associations with hypoxia and proliferation markers, further supporting the feasibility and effectiveness of non-invasive PET/CT monitoring in tumor radiotherapy.

New therapeutic strategies, especially dose escalation to the hypoxic area, is expected to improve the prognosis of patients. Thus, the evaluation of dynamic changes in intratumoral hypoxic and proliferative states by a dual-tracer study illuminates the understanding of tumor biology after radiotherapy, thereby facilitating its planning, including dose escalation and altered fraction schedules. Dose escalation requires an accurate determination of the hypoxic area in order to target the hypoxia. New developed PET scanners with high spatial resolution and the new generation of hypoxic tracers with lower lipid solubility [for example, ^18^F-fluoroazomycin arabinoside (^18^F-FAZA)] would have the potential to accurately demarcate the hypoxic area with high contrast (37).

This study was limited by its small sample size. In addition, the survival rate and prognosis of reoxygenated tumors after radiotherapy were not assessed. Additional research is needed to confirm these findings, especially studies involving patients.

## Conclusion

Reoxygenation of hypoxic tumors occurs early after radiotherapy initiation. In contrast, cell proliferation inhibition associated with tumoricidal effects takes place gradually with the course of radiotherapy. Finally, ^18^F-FMISO and ^18^F-FLT PET/CT are sensitive and non-invasive tools for monitoring tumor reoxygenation and activity during fractionated radiotherapy.

## Data Availability Statement

All datasets generated for this study are included in the article/supplementary material.

## Ethics Statement

The animal studies were reviewed and approved by the Institute of Anhui Medical University, and followed AAALAC and IACUC guidelines.

## Author Contributions

WY and HX designed the experiment and wrote the paper. WY, XS, DZ, and FQ performed the experiment and statistical analysis of data. HW and JJ contribute technical help. All authors have read and approved this version of the paper.

## Conflict of Interest

The authors declare that the research was conducted in the absence of any commercial or financial relationships that could be construed as a potential conflict of interest.

## Abbreviations

^18^F-FLT: ^18^F-fluorothymidine
^18^F-FMISO: F-fluoromisonidazole
PET/CT: positron emission tomography/computed tomography.

## References

[1] Kolesnik DL Pyaskovskaya ON Boichuk IV Solyanik GI. Hypoxia enhances antitumor activity of dichloroacetate. Exp Oncol. (2014) 36:231–5. 25537215

[2] Brown JM Therapeutic targets in radiotherapy. Int J Radiat Oncol Biol Phys. (2001) 49:319–26. 10.1016/S0360-3016(00)01482-6 11173124

[3] Flynn JR Wang L Gillespie DL Stoddard GJ Reid JK Owens J. Hypoxia-regulated protein expression, patient characteristics, and preoperative imaging as predictors of survival in adults with glioblastoma multiforme. Cancer. (2008) 113:1032–42. 10.1002/cncr.23678 18618497 PMC2574798

[4] Evans SM Jenkins KW Chen HI Jenkins WT Judy KD Hwang WT. The relationship among hypoxia, proliferation, and outcome in patients with *de novo* glioblastoma: a Pilot study. Transl Oncol. (2010) 3:160–9. 10.1593/tlo.09265 20563257 PMC2887645

[5] Nordsmark M Bentzen SM Rudat V Brizel D Lartigau E Stadler P. Prognostic value of tumor oxygenation in 397 head and neck tumors after primary radiation therapy. An international multi-center study. Radiother Oncol. (2005) 77:18–24. 10.1016/j.radonc.2005.06.038 16098619

[6] Harriss W Bezak E Yeoh E Hermans M. Measurement of reoxygenation during fractionated radiotherapy in head and neck squamous cell carcinoma xenografts. Australas Phys Eng Sci Med. (2010) 33:251–63. 10.1007/s13246-010-0032-6 20878297

[7] Wenzl T Wilkens JJ. Theoretical analysis of the dose dependence of the oxygen enhancement ratio and its relevance for clinical applications. Radiat Oncol. (2011) 6:171. 10.1186/1748-717X-6-171 22172079 PMC3283483

[8] Bernsen HJ Rijken PF Peters H Raleigh JA Jeuken JW Wesseling P. Hypoxia in a human intracerebral glioma model. J Neurosurg. (2000) 93:449–54. 10.3171/jns.2000.93.3.0449 10969943

[9] Rasey JS Koh WJ Evans ML Peterson LM Lewellen TK Graham MM. Quantifying regional hypoxia in human tumors with positron emission tomography of [18F]fluoromisonidazole: a pretherapy study of 37 patients. Int J Radiat Oncol Biol Phys. (1996) 36:417–28. 10.1016/S0360-3016(96)00325-2 8892467

[10] Hatano T Zhao S Zhao Y Nishijima K Kuno N Hanzawa H. Biological characteristics of intratumoral [F-18]fluoromisonidazole distribution in a rodent model of glioma. Int J Oncol. (2013) 42:823–30. 10.3892/ijo.2013.1781 23338175 PMC3597456

[11] Wang H Zhang Y Yu W Xue Y Xiao L Xu H. Hypoxia imaging and biological evaluation of the radiosensitizing effect of oleanolic acid. Biomed Res Int. (2018) 2018:2694679. 10.1155/2018/2694679 30246018 PMC6136542

[12] Thorwarth D Eschmann SM Paulsen F Alber M. A model of reoxygenation dynamics of head-and-neck tumors based on serial 18F-fluoromisonidazole positron emission tomography investigations. Int J Radiat Oncol Biol Phys. (2007) 68:515–21. 10.1016/j.ijrobp.2006.12.037 17398015

[13] Eschmann SM Paulsen F Bedeshem C Machulla HJ Hehr T Bamberg M. Hypoxia-imaging with (18)F-Misonidazole and PET: changes of kinetics during radiotherapy of head-and-neck cancer. Radiother Oncol. (2007) 83:406–10. 10.1016/j.radonc.2007.05.014 17543402

[14] Dirix P Vandecaveye V De Keyzer F Stroobants S Hermans R Nuyts S. Dose painting in radiotherapy for head and neck squamous cell carcinoma: value of repeated functional imaging with (18)F-FDG PET, (18)F-fluoromisonidazole PET, diffusion-weighted MRI, and dynamic contrast-enhanced MRI. J Nucl Med. (2009) 50:1020–7. 10.2967/jnumed.109.062638 19525447

[15] Narita T Aoyama H Hirata K Onodera S Shiga T Kobayashi H. Reoxygenation of glioblastoma multiforme treated with fractionated radiotherapy concomitant with temozolomide: changes defined by 18F-fluoromisonidazole positron emission tomography: two case reports. Jpn J Clin Oncol. (2012) 42:120–3. 10.1093/jjco/hyr181 22198964

[16] Tachibana I Nishimura Y Shibata T Kanamori S Nakamatsu K Koike R. A prospective clinical trial of tumor hypoxia imaging with 18F-fluoromisonidazole positron emission tomography and computed tomography (F-MISO PET/CT) before and during radiation therapy. J Radiat Res. (2013) 54:1078–84. 10.1093/jrr/rrt033 23589026 PMC3823770

[17] Okamoto S Shiga T Yasuda K Watanabe S Hirata K Nishijima KI. The reoxygenation of hypoxia and the reduction of glucose metabolism in head and neck cancer by fractionated radiotherapy with intensity-modulated radiation therapy. Eur J Nucl Med Mol Imaging. (2016) 43:2147–54. 10.1007/s00259-016-3431-4 27251644

[18] Shields AF Grierson JR Dohmen BM Machulla HJ Stayanoff JC Lawhorn-Crews JM. Imaging proliferation *in vivo* with [F-18]FLT and positron emission tomography. Nat Med. (1998) 4:1334–6. 10.1038/3337 9809561

[19] Lee TS Ahn SH Moon BS Chun KS Kang JH Cheon GJ. Comparison of 18F-FDG, 18F-FET and 18F-FLT for differentiation between tumor and inflammation in rats. Nucl Med Biol. (2009) 36:681–6. 10.1016/j.nucmedbio.2009.03.009 19647174

[20] van Waarde A Jager PL Ishiwata K Dierckx RA Elsinga PH. Comparison of sigma-ligands and metabolic PET tracers for differentiating tumor from inflammation. J Nucl Med. (2006) 47:150–4. 16391199

[21] Okamoto S Shiga T Yasuda K Ito YM Magota K Kasai K. High reproducibility of tumor hypoxia evaluated by 18F-fluoromisonidazole PET for head and neck cancer. J Nucl Med. (2013) 54:201–7. 10.2967/jnumed.112.109330 23321456

[22] Yue J Chen L Cabrera AR Sun X Zhao S Zheng F. Measuring tumor cell proliferation with 18F-FLT PET during radiotherapy of esophageal squamous cell carcinoma: a pilot clinical study. J Nucl Med. (2010) 51:528–34. 10.2967/jnumed.109.072124 20237030

[23] Shibamoto Y Iwata H. The quest for optimal fractionation schedules in stereotactic radiotherapy. Cureus. (2020) 12:e6777. 10.7759/cureus.6777 32117663 PMC7041652

[24] Forster JC Marcu LG Bezak E. Approaches to combat hypoxia in cancer therapy and the potential for *in silico* models in their evaluation. Phys Med. (2019) 64:145–56. 10.1016/j.ejmp.2019.07.006 31515013

[25] Marcu LG Marcu D. The role of hypofractionated radiotherapy in the management of head and neck cancer - a modelling approach. J Theor Biol. (2019) 482:109998. 10.1016/j.jtbi.2019.109998 31493484

[26] Ljungkvist AS Bussink J Kaanders JH Wiedenmann NE Vlasman R van der Kogel AJ. Dynamics of hypoxia, proliferation and apoptosis after irradiation in a murine tumor model. Radiat Res. (2006) 165:326–36. 10.1667/RR3515.1 16494521

[27] Bussink J Kaanders JH Rijken PF Raleigh JA Van der Kogel AJ. Changes in blood perfusion and hypoxia after irradiation of a human squamous cell carcinoma xenograft tumor line. Radiat Res. (2000) 153:398–404. 10.1667/0033-7587(2000) 153[0398:CIBPAH]2.0.CO;2 10760999

[28] Crokart N Jordan BF Baudelet C Ansiaux R Sonveaux P Gregoire V. Early reoxygenation in tumors after irradiation: determining factors and consequences for radiotherapy regimens using daily multiple fractions. Int J Radiat Oncol Biol Phys. (2005) 63:901–10. 10.1016/j.ijrobp.2005.02.038 16199320

[29] Xu H Sun G Wang H Yue Q Tang H Wu Q. Dynamic observation of the radiosensitive effect of irisquinone on rabbit VX2 lung transplant tumors by using fluorine-18-deoxyglucose positron emission tomography/computed tomography. Nucl Med Commun. (2013) 34:220–8. 10.1097/MNM.0b013e32835d3730 23276827

[30] Fei M Guan J Xue T Qin L Tang C Cui G. Hypoxia promotes the migration and invasion of human hepatocarcinoma cells through the HIF-1alpha-IL-8-Akt axis. Cell Mol Biol Lett. (2018) 23:46. 10.1186/s11658-018-0100-6 30258464 PMC6149064

[31] Teppo S Sundquist E Vered M Holappa H Parkkisenniemi J Rinaldi T. The hypoxic tumor microenvironment regulates invasion of aggressive oral carcinoma cells. Exp Cell Res. (2013) 319:376–89. 10.1016/j.yexcr.2012.12.010 23262025

[32] Perez-Sayans M Suarez-Penaranda JM Pilar GD Supuran CT Pastorekova S Barros-Angueira F. Expression of CA-IX is associated with advanced stage tumors and poor survival in oral squamous cell carcinoma patients. J Oral Pathol Med. (2012) 41:667–74. 10.1111/j.1600-0714.2012.01147.x 22486898

[33] Jubb AM Buffa FM Harris AL. Assessment of tumour hypoxia for prediction of response to therapy and cancer prognosis. J Cell Mol Med. (2010) 14:18–29. 10.1111/j.1582-4934.2009.00944.x 19840191 PMC3837600

[34] Chen J Rocken C Hoffmann J Kruger S Lendeckel U Rocco A. Expression of carbonic anhydrase 9 at the invasion front of gastric cancers. Gut. (2005) 54:920–7. 10.1136/gut.2004.047340 15951534 PMC1774603

[35] Zinczuk J Zareba K Guzinska-Ustymowicz K Kedra B Kemona A Pryczynicz A. Expression of chosen cell cycle and proliferation markers in pancreatic intraepithelial neoplasia. Prz Gastroenterol. (2018) 13:118–26. 10.5114/pg.2018.75824 30002770 PMC6040105

[36] Morgunova E Gray FC Macneill SA Ladenstein R. Structural insights into the adaptation of proliferating cell nuclear antigen (PCNA) from Haloferax volcanii to a high-salt environment. Acta Crystallogr D Biol Crystallogr. (2009) 65(Pt 10):1081–8. 10.1107/S0907444909029321 19770505 PMC2756170

[37] Halmos GB Bruine de Bruin L Langendijk JA van der Laan BF Pruim J Steenbakkers RJ. Head and neck tumor hypoxia imaging by 18F-fluoroazomycin-arabinoside (18F-FAZA)-PET: a review. Clin Nucl Med. (2014) 39:44–8. 10.1097/RLU.0000000000000286 24152663

